# Tube-Wise Diagnostic Microarray for the Multiplex Characterization of the Complex Plant Pathogen *Ralstonia solanacearum*

**DOI:** 10.3389/fpls.2017.00821

**Published:** 2017-05-24

**Authors:** Gilles Cellier, Sandrine Arribat, Frédéric Chiroleu, Philippe Prior, Isabelle Robène

**Affiliations:** ^1^Tropical Pests and Diseases Unit, Plant Health Laboratory, ANSESSaint-Pierre, France; ^2^UMR Peuplements Végétaux et Bioagresseurs en Milieu Tropical, CIRADSaint-Pierre, France; ^3^Département Santé des Plantes et Environnement, Institut National de la Recherche AgronomiqueSaint-Pierre, France

**Keywords:** *Ralstonia solanacearum*, microarray, ArrayTube, multiplexing, diagnostic

## Abstract

*Ralstonia solanacearum* is a well-known agricultural and ecological threat worldwide. The complexity of the *R. solanacearum* species complex (Rssc) represents a challenge for the accurate characterization of epidemiological strains by official services and research laboratories. The majority of protocols only focus on a narrow range of strains; however, this species complex includes strains that represent major constraints and are under strict regulation. The main drawback associated with the current methods of detecting and characterizing Rssc strains is their reliance on combining different protocols to properly characterize the strains at the ecotype level, which require time and money. Therefore, we used microarray technology (ArrayTube) to develop a standard protocol, which characterizes 17 major groups of interest in the Rssc, in a single multiplex reaction. These 17 majors groups are linked with a phylogenetic assignation (phylotypes, sequevars), but also with an ecotype assignation associated with a range of hosts (e.g., brown rot, Moko). Probes were designed with a 50-mer length constraint and thoroughly evaluated for any flaws or secondary structures. The strains are characterized based on a DNA extraction from pure culture. Validation data showed strong intra-repeatability, inter-repeatability, and reproducibility as well as good specificity. A hierarchical analysis of the probe groups is suitable for an accurate characterization. Compared with single marker detection tests, the method described in this paper addresses efficiently the issue of combining several tests by testing a large number of phylogenetic markers in a single reaction assay. This custom microarray (RsscAT) represents a significant improvement in the epidemiological monitoring of Rssc strains worldwide, and it has the potential to provide insights for phylogenetic incongruence of Rssc strains based on the host of isolation and may be used to indicate potentially emergent strains.

## Introduction

Bacterial strains of the *Ralstonia solanacearum* species complex (Rssc) are considered priority plant pathogens in many countries in the world and are classified as quarantine organisms as well as bioterrorism and double usage agents in the USA and in Europe (2000; 2008). Plant protection across borders and territories requires the availability of rapid and reliable protocols to prevent the accidental or intentional introduction of exotic strains. The genetic diversity of Rssc strains is distributed into four major phylotypes and 53 sequevars based on partial sequences of the endoglucanase *(egl)* gene (Fegan and Prior, 2005). The number of sequevars or lineages can be extended with the discovery of new strains, as demonstrated in sequevars that were identified and characterized in Martinique (French West Indies; Wicker et al., 2009) and Brazil (Albuquerque et al., 2014). A recent polyphasic taxonomic approach based primarily on DNA-DNA hybridizations, Internal transcribed spacer (ITS) and *egl* partial sequence phylogenetic analyses, and phenotypic data merged the Rssc into three species (Safni et al., 2014): *R. solanacearum* clustering strains from phylotype II; *R. pseudosolanacearum* clustering strains from phylotypes I and III; and *R. syzygii* subsp. *syzygii* comb. nov. clustering strains from phylotype IV, which included two sub-species, *R. syzygii* subsp. *celebesensis* subsp. nov. and *R. syzygii* subsp. *indonesiensis* subsp. nov. These data were supported by comparative analyses of whole bacterial genomes (Prior et al., 2016).

Such complexity represents a challenge for any diagnostician attempting to develop protocols and tools to specifically discriminate Rssc strains. Moreover, the ability to identify and distinguish Rssc ecotypes, a group of bacteria sharing the same ecological niche (Cohan, 2002), is important because they represent the most epidemiological active strains in the Rssc causing “famous” diseases, such as potato brown rot or the banana Moko, in valuable agronomic plants. Certain ecotypes can be split into different phylogenetic groups, such as the paraphyletic Moko strains pathogenic to *Musaceae* and *Solanaceae* (Cellier et al., 2012; Albuquerque et al., 2014), which have been phylogenetically categorized into sequevars 3, 4, 6, 24, 25, 41, and 53. Moreover, the difficulty of discriminating among ecotypes increases when high proximity is observed in the genome content of the ecotypes, which has been observed for the pathological variant IIB-4NPB strains (not pathogenic on banana) and Moko sequevar 4 strains (Cellier et al., 2012; Ailloud et al., 2015).

Diagnostic protocols mainly rely on techniques that offer compatible routine application procedures and provide for user-friendly environments, good industrial production capacities, and a high level of reliability. Techniques that primarily employ immunoassays and immune-strip tests (ImmunoStrip®; Agdia, Elkhart, IN) are mainly used for the detection of Rssc strains at the species level (Danks and Barker, 2000), and although these assays are simple and affordable, they are known to produce false positives (Narayanasamy, 2010). DNA-based approaches using conventional PCR (Table 1) can identify the Rssc at the species level (Huang and Schell, 1990; Seal et al., 1993; Opina et al., 1997; Lee and Wang, 2000; Glick et al., 2002; Schonfeld et al., 2003), the phylotype level (Fegan and Prior, 2005); and when these approaches are coupled with sequencing capabilities, they can be used to produce phylogenetic trees (Lane, 1991; Taghavi et al., 1996; Fegan and Prior, 2005; Prior and Fegan, 2005b). General protocols that rely on the evolution of PCR techniques are also available, such as qPCR (Weller et al., 2000; Ozakman and Schaad, 2003; Smith and De Boer, 2009; Inoue and Nakaho, 2014) or LAMP PCR (Lenarcic et al., 2014). Specific protocols have been designed to target a particular group of strains or ecotypes of the Rssc, such as brown rot strains (Fegan et al., 1998; Weller et al., 2000; Ozakman and Schaad, 2003; Smith and De Boer, 2009; Kubota et al., 2011; Ha et al., 2012; Li et al., 2014; Kubota and Jenkins, 2015; Stulberg et al., 2015); Moko strains (Prior and Fegan, 2005a; Cellier et al., 2015); and Blood Disease Bacterium (BDB) strains (Kubota et al., 2011). These diagnostic methods are primarily limited to the detection of strains associated with the brown rot ecotype (Li et al., 2014); however, the Rssc presents a wide diversity of strains.

**Table 1:** **List of available PCR tools for the ***Ralstonia solanacearum*** species complex diagnostic**.

**PCR names**	**Phylogenetic group**	**Primers name**	**Target**	**Primer sequence**	**bp**	**Bibliographic References**
Phylotypes Multiplex	I	Nmult:21:1F	ITS	CGTTGATGAGGCGCGCAATTT	144	Fegan and Prior, 2005
	II	Nmult:21:2F		AAGTTATGGACGGTGGAAGTC	372	
	III	Nmult:23:AF		TTACSAGAGCAATCGAAAGATT	91	
	IV	Nmult:22:InF		ATTGCCAAGACGAGAGAAGTA	213	
	na	Nmult:22:RR		TCGCTTGACCCTATAACGAGTA	na	
Moko Multiplex	Seq 3	MUS35-F	Uncharacterized	GCAGTAAAGAAACCCGGTGTT	401	Prior and Fegan, 2005a
		MUS35-R		TCTGGCGAAAGACGGGATGG		
	Seq 3	IS24-F	ISRso19	TCGGGCGTGAAGAGGCAGAC	490	Bagsic-Opulencia et al., 2006
		IS24-R		GGAGGTGTGCGCCATCAACTG		
	Seq 4	MUS20-F	*RhiG*	CGGGTGGCTGAGACGAATATC	351	Prior and Fegan, 2005a
		MUS20-R		GCCTTGTCCAGAATCCGAATG		
	Seq 4 **PB**	MUS06-F	Uncharacterized	GCTGGCATTGCTCCCGCTCAC	167	Prior and Fegan, 2005a
		MUS06-R		TCGCTTCCGCCAAGACGC		
	Seq 6	SI28-F	Uncharacterized	CGTTCTCCTTGTCAGCGATGG	220	Prior and Fegan, 2005a
		SI28-R		CCCGTGTGACCCCGATAGC		
	Seq 24	VC46-F	Uncharacterized	CTCCTGGGAGTCGGTTGGGTC	100	Woo et al., unpublished
		VC46-R		AGGGAACCTAGGCGTGACTG		
*IpxC*	*Rssc*	759	*IpxC*	GTCGCCGTCAACTCACTTTCC	282	Opina et al., 1997
		760		GTCGCCGTCAGCAATGCGGAATCG		
*pehA*	*Rssc*	pehA 3	*pehA*	CAGCAGAACCCGCGCCTGATCCAG	480	Huang and Schell, 1990
		pehA 6		ATCGGACTTGATGCGCAGGCCGTT		
*pehB*	*Rssc*	RS3	*pehB*	AGCACGACCGGTGCGACCTGCT	822	Glick et al., 2002
		RS4		CACCCCGCGCGTGTCGTCGTAG		
*fliC*	*Rssc*	fliC F	*fliC*	GAACGCCAACGGTGCGAACT	400	Schonfeld et al., 2003
		fliC R		GGCGGCCTTCAGGGAGGTC		
*BP4*	*Rssc*	BP4-R	Unknown—Cloned RAPD fragment	GACGACATCATTTCCACCGGGCG	1102	Lee and Wang, 2000
		BP4-L		GGGTGAGATCGATTGTCTCCTTG		
16S rRNA	*Rssc*	OLI1	16S	GGGGGTAGCTTGCTACCTGCC	288	Seal et al., 1993
		Y2		CCCACTGCTGCCTCCCGTAGGAGT		
*mutS*	*Rssc*	mutS-RsF.1570	*mutS*	ACAGCGCCTTGAGCCGGTACA	758	Prior and Fegan, 2005a
		mutS-RsR.1926		GCTGATCACCGGCCCGAACAT		
*egl*	*Rssc*	Endo-R	*egl*	GCGTTGCCCGGCACGAACACC	800	Fegan and Prior, 2005
		Endo-F		ATGCATGCCGCTGGTCGCCGC		
16S rRNA	*Rssc*	27F	*16S*	AGAGTTTGATMTGGCTCAG	48	Taghavi et al., 1996
		1492R		GGTTACCTTGTTACGACTT		
16S-23S rRNA ITS region	*Rssc*	1100F	ITS	GCAACGAGCGCAACCC	50	Lane, 1991
		240R		TTCGCTCGCCACTACT		
16S-23S rRNA ITS region	*Rssc*	L1	ITS	AGTCGTAACAAGGTAGCCG	48	Fegan et al., 1998
		PS-23Sr		TACTACGTCCTTCATCG		
Brown rot	Seq1 & Seq2	630	Genomic DNA fragment “prophage region”	ATACAGAATTCGACCGGCACG	307	Fegan et al., 1998
		631		AATCACATGCAATTCGCCTACG		
IIB-4NPB	IIB-4NPB	5F	Genomic DNA fragment	GCGCGCGAGGCTGGTGATGT	661	Cellier et al., 2015
		5R		TGGGTTCGCAGGCGGACAGC		
Moko	Moko & NPB	93F	*KfrA*	CGCTGCGCGGCCGTTTCAC	477	Cellier et al., 2015
		93R		CGGTCGCGGCATGGGCTT		
BDB	BDB	121F	Uncharacterized	CGTATTGGATGCCGTAATGGA	344	Tan, 2003
		121R		AAGTTCATTGGTGCCGAATCA		
BDB	BDB	BDB2400-F	*GpS*	GCTGACTATAGGCACAGCGG	131	Kubota et al., 2011
		BDB2400-R		AATCGCCGTTCCCATACAAG		

The main drawback associated with the current methods of detecting and characterizing Rssc strains relies in combining different protocols to properly characterize the strains at their ecotype level. The development of a single method to fully characterize the main groups/ecotypes of the Rssc would greatly benefit the diagnostic field and streamline the workflow for strain identification in an efficient and affordable manner, and it would also facilitate the detection of emerging or unknown strains.

In this study, we employed a multiplex approach to manage the complexity of the Rssc within the framework of microarray technology, which has the potential to test for multiple biomolecular targets in a single reaction. In recent years, applications for the Rssc have been developed for both fundamental (Guidot et al., 2007, 2009; Cellier et al., 2012; Lefeuvre et al., 2013) and applied research (Aittamaa et al., 2008; Pelludat et al., 2009; Dobnik et al., 2014). To provide a cost-effective strategy that meets the need for user-friendly processing via conventional lab equipment and high-volume manufacturing capacities that comply with *in vitro* diagnostic (IVD) regulations, the ArrayTube (AT) platform (Alere Technologies GmbH, Jena, Germany) was selected as the final protocol. This array consists of a custom microarray integrated into a micro reaction vial, which simplifies handling and is used for routing testing in the medical field (Braun et al., 2012; Schneeberg et al., 2015).

Recently, new genomes of the Rssc were fully sequenced and annotated (Ailloud et al., 2015), thus providing new insights for the development of more reliable markers for diagnostic purposes. This paper provides the first description of the production of a microarray (RsscAT) dedicated to characterize strains within 17 major groups of interest in the Rssc with respect to their phylogenetic assignation and ecotype. This portable custom RsscAT provides significant improvements that allow for the rapid assignment of the phylogenetic position of a strain, which can be used to predict the pathogenicity of a strain. RsscAT has the potential to be used for the epidemiological monitoring of heterogeneous Rssc strains worldwide.

## Materials and methods

### Bacterial strains

A set of 75 bacterial reference strains (Table 2) was selected to cover the known genetic diversity among Rssc strains. Another set of 12 outgroup strains (Table 2) was used to assess the specificity of each designed probe, It contained strains that are known to be isolated from *Solanaceae, Musaceae*, soil, water, and strains that are phylogenetically closely related to the Rssc. The bacterial strains were obtained from the *Centre de coopération Internationale en Recherche Agronomique pour le Développement* (CIRAD—Saint Pierre, Reunion Island), and they were stored at −80°C on cryobeads (Microbank, Pro-labs Diagnostics, Toronto, Canada). The bacteria were cultured overnight in Luria-Bertani broth (LB) at 28°C with agitation at 250 rpm. The Rssc strains were streaked on modified Sequeira semi-selective solid medium containing agar (18 g/L), yeast extract (1 g/L), peptone (11 g/L), glycerol (6.3 g/L), crystal violet (2 mg/L), polymyxin-β-sulfate (10 mg/L), tyrothricine (20 mg/L), chloramphenicol (5 mg/L), 2,3,5-triphenyltetrazolium chloride (11 mg/L), Tilt (Propiconazole; Syngenta, Bβle, Switzerland; 0.004%), and penicillin (20 U) and then incubated for 48 h at 28°C. The 12 outgroup strains were streaked on LPGA solid medium containing agar (18 g/L), yeast extract (7 g/L), peptone (7 g/L), and glucose (10 g/L) and then incubated for 24 h at 28°C.

**Table 2:** *****Ralstonia solanacearum*** strains and outgroup strains used for validating the RsscAT**.

**Strain ID**	**World**	**Country**	**Year**	**Host**	**Phylotype**	**Sequevar**	**Group**		
02-204	West Indies	Martinique	2002	*Solanum lycopersicum*	IIB	4NPB	II	IIB	IIB4NPB
447	Indian Ocean	Madagascar	2013	*Solanum tuberosum*	III	na	III		
594	Indian Ocean	Madagascar	2013	*Solanum tuberosum*	III	na	III		
6039	America (South)	French Guiana	2006	Water (irrigation)	IIB	4NPB	II	IIB	IIB4NPB
A3909	America (North)	USA	1989	*Heliconia rostrata*	IIA	6	II	IIA	IIA6
AP31H	America (South)	Uruguay	2003	*Solanum tuberosum*	IIB	1	II	IIB	IIB1-2
B26	America (South)	Brazil	1997	*Musa* sp.	IIA	24	II	IIA	IIA24
B34	America (South)	Brazil	1998	*Musa* sp.	IIA	24	II	IIA	IIA24
B50	America (South)	Brazil	1998	*Musa* sp.	IIA	24	II	IIA	IIA24
B91	America (South)	Brazil	2000	*Musa* sp.	IIA	24	II	IIA	IIA24
CFBP1410	America (South)	Colombia	1997	Banana plantain	IIB	2	II	IIB	IIB1-2
CFBP1416	America (Central)	Costa Rica	1997	Banana plantain	IIB	3	II	IIB	IIB3
CFBP2047	America (North)	USA	1953	*Solanum lycopersicum*	IIA	7	II	IIA	IIA7
CFBP2957	West Indies	Martinique	1987	*Solanum lycopersicum*	IIA	36	II	IIA	
CFBP2958	West Indies	Guadeloupe	1985	*Solanum lycopersicum*	IIA	39	II	IIA	
CFBP3858	Europe (North)	Netherlands	1995	*Solanum tuberosum*	IIB	1	II	IIB	IIB1-2
CFBP3879	America (South)	Colombia	1992	*Solanum tuberosum*	IIB	2	II	IIB	IIB1-2
CFBP4801	Indian Ocean	Reunion	1988	*Solanum lycopersicum*	IIB	1	II	IIB	IIB1-2
CFBP4963	Indian Ocean	Reunion	na	*Solanum tuberosum*	III	19	III		
CFBP4964	Indian Ocean	Reunion	1994	*Pelargonium asperum*	III	19	III		
CFBP6727	Asia	Indonesia	na	*Solanum tuberosum*	IV	10	IV		RsIV
CFBP6783	West Indies	Martinique	2002	*Heliconia caribea*	IIB	4NPB	II	IIB	IIB4NPB
CFBP6797	West Indies	Martinique	2002	*Solanum americanum*	IIB	4NPB	II	IIB	IIB4NPB
CFBP7058	Africa	Cameroon	2005	*Solanum scabrum*	I	13	I		
CIP239	America (South)	Brazil	1983	*Solanum tuberosum*	IIA	40	II	IIA	
CIP365	Asia	Philippines	1989	*Solanum tuberosum*	I	45	I		
CIP417	Asia	Philippines	1991	*Musa* sp.	IIB	3	II	IIB	IIB3
CIV30	Africa	Ivory Coast	2010	*Solanum lycopersicum*	IIA	35	II	IIA	
CMR15	Africa	Cameroon	2005	*Solanum lycopersicum*	III	29	III		
CMR33	Africa	Cameroon	2005	*Solanum lycopersicum*	III	20	III		
DGBBC1138	Africa	Guinea	na	*Solanum tuberosum*	III	43	III		
ETAC	America (South)	Uruguay	2004	*Solanum tuberosum*	IIB	1	II	IIB	IIB1-2
GMI1000	America (South)	French Guiana	1978	*Solanum lycopersicum*	I	18	I		
GMI8044	West Indies	Grenada	1984	Banana	IIA	6	II	IIA	IIA6
GMI8254	Asia	Indonesia	na	*Solanum lycopersicum*	I	47	I		
Grenada 9-1	West Indies	Grenada	2007	Banana bluggoe	IIA	6	II	IIA	IIA6
GUY B06E2	America (South)	Guyana	2008	*Musa acuminata × balbisiana aaa*	IIA	6	II	IIA	IIA6
IBSBF1503	America (South)	Brazil	1999	*Cucumis sativus*	IIB	4NPB	II	IIB	IIB4NPB
IBSBF1900	America (South)	Brazil	2000	*Musa* sp.	IIA	24	II	IIA	IIA24
IMI370184	America (Central)	Costa-Rica	1996	*Musa* sp.	IIB	3	II	IIB	IIB3
IPO1609	Europe (North)	Netherlands	1995	*Solanum tuberosum*	IIB	1	II	IIB	IIB1-2
JQ1006	Indian Ocean	Reunion	1993	*Solanum tuberosum*	IIB	1	II	IIB	IIB1-2
JQ1143	Indian Ocean	Reunion	na	*Solanum tuberosum*	IIA	39	II	IIA	
JT511	Indian Ocean	Reunion	1993	*Solanum tuberosum*	IIB	1	II	IIB	IIB1-2
JT516	Indian Ocean	Reunion	1993	*Solanum tuberosum*	IIB	1	II	IIB	IIB1-2
JT525	Indian Ocean	Reunion	1993	*Pelargonium asperum*	III	19	III		
JT644	America (Central)	Costa Rica	1998	*Heliconia rostrata*	IIB	3	II	IIB	IIB3
JT663	Asia	Indonesia	1998	*Syzygium aromaticum*	IV	9A	IV		SZY
JY200	West Indies	Martinique	1999	*Anthurium andreanum*	IIB	4NPB	II	IIB	IIB4NPB
LNPV24.25	Europe (North)	France	2001	*Solanum lycopersicum*	IIB	4NPB	II	IIB	IIB4NPB
LNPV28.23	Indian Ocean	Reunion	2004	*Solanum tuberosum*	IIB	1	II	IIB	IIB1-2
MAFF301558	Asia	Japan	2006	*Solanum tuberosum*	IV	8	IV		RsIV
MG144	Indian Ocean	Madagascar	2013	*Solanum tuberosum*	II	1	II	IIB	IIB1-2
MG27	Indian Ocean	Madagascar	2013	*Solanum tuberosum*	III	19	III		
MG464	Indian Ocean	Madagascar	2013	*Solanum tuberosum*	III	19	III		
MG49	Indian Ocean	Madagascar	2013	*Solanum tuberosum*	II	1	II	IIB	IIB1-2
MG713	Indian Ocean	Madagascar	2013	*Solanum tuberosum*	II	1	II	IIB	IIB1-2
MG732	Indian Ocean	Madagascar	2013	*Solanum tuberosum*	II	1	II	IIB	IIB1-2
MG837	Indian Ocean	Madagascar	2013	*Solanum tuberosum*	II	1	II	IIB	IIB1-2
MG85	Indian Ocean	Madagascar	2013	*Solanum tuberosum*	III	na	III		
MOLK2	Asia	Philippines	1991	*Musa* sp.	IIB	3	II	IIB	IIB3
NCPPB1018	Africa	Angola	1961	*Solanum tuberosum*	III	21	III		
PSI07	Asia	Indonesia	na	*Solanum lycopersicum*	IV	10	IV		RsIV
PSS4	Asia	Taiwan	1988	*Solanum lycopersicum*	I	15	I		
R229	Asia	Indonesia	1988	Banana	IV	10	IV		BDB
R24	Asia	Indonesia	na	*Syzygium aromaticum*	IV Hr+	9B	IV		SZY
R28	Asia	Indonesia	na	*Syzygium aromaticum*	IV	9	IV		SZY
RF32	West Indies	Trinidad	2003	*Solanum lycopersicum*	IIA	7	II	IIA	IIA7
UQRS283	Asia	Indonesia	na	*Solanum lycopersicum*	IV	10	IV		RsIV
UQRS627	Asia	Indonesia	2005	*Musa* sp.	IV	10	IV		BDB
UQRS633	Asia	Indonesia	2005	*Musa* sp.	IV	10	IV		BDB
UW163	America (South)	Peru	1967	Banana plantain	IIB	4	II	IIB	IIB4
UW179	America (South)	Colombia	1961	Banana plantain	IIB	4	II	IIB	IIB4
UW181	America (South)	Venezuela	1960	Banana plantain	IIA	6	II	IIA	IIA6
UW551	Africa	Kenya	2003	*Pelargonium asperum*	IIB	1	II	IIB	IIB1-2
CFBP7122	Africa	Ethiopia	1995	*Musa* sp.	*Xanthomonas vasicola* pv. *musacearum*				
LMG0911	Oceania	New Zealand	1957	*Solanum lycopersicum*	*Xanthomonas vesicatoria*				
LMG1199	America (North)	USA	1957	Soil	*Cupriavidus necator*				
LMG16206	na	na	1995	na	*Pseudomonas putida*				
LMG1794	Europe (North)	United Kingdom	1951	Water (irrigation)	*Pseudomonas fluorescens*				
LMG2172	Europe (North)	United Kingdom	1972	*Solanum lycopersicum*	*Pseudomonas corrugata*				
LMG2804	America (North)	USA	1956	*Chrysanthemum morifolium*	*Dickeya chrysanthemi* bv. *chrysanthemi*				
LMG2894	Europe (North)	Sweden	1956	*Solanum tuberosum*	*Clavibacter michiganensis* subsp. *sepedonicus*				
LMG5093	Europe (North)	United Kingdom	1960	*Solanum lycopersicum*	*Pseudomonas syringae* pv. *tomato*				
LMG5942	America (North)	USA	1974	Human	*Ralstonia pickettii*				
LMG7333	Europe (North)	Hungary	1957	*Solanum lycopersicum*	*Clavibacter michiganensis* subsp. *michiganensis*				
NCPPB2968	America (North)	USA	1977	*Capsicum frutescens*	*Xanthomonas axonopodis* pv. *vesicatoria*				

The groups of interest were sorted to resemble the phylogeny of the Rssc (phylotypes, ecotypes) and to reflect epidemic events that have occurred worldwide and that have been reported in the literature. Based on the classification in sequevars, we identified 17 groups (Table 3), including the 4 phylotypes; brown rot strains (sequevars 1 and 2); Moko strains (sequevars 3, 4, 6, and 24), epidemiological variant 4NPB, Grandville wilt strains (sequevar 7); *R. syzygii* subsp. *indoniesensis* and *R. syzygii* subsp. *celebensis* (BDB).

**Table 3:** **The 17 groups of interest of the ***Ralstonia solanacearum*** species complex**.

**Group of interest**	**Phylotype**	**Sequevars**	**Common name**	**nb Hyb strains^c^**
GC	All	All known sequevars 1–53	Rssc	128
I	I	All Phylotype I sequevars: 12;13;14;15;16;17;18;31;34;44;45;46;47;48		7
II	II	All Phylotype II sequevars: 1;2;3;4;4NPB;5;6;7;24;25;26;27;28;28;35;36;37;38;39;40;41;50;51;52;53		80
IIA	IIA	All Phylotype IIA sequevars: 5;6;7;24;28;35;36;37;38;39;40;41;50;52;53		30
IIA6	IIA	6	Moko	9
IIA7	IIA	7	Grandville wilt	7
IIA24	IIA	24	Moko	7
IIB	IIB	All Phylotype IIB sequevars: 1;2;3;4;4NPB;25;26;27;28;51		50
IIB1-2	IIB	1;2	Brown rot	24
IIB3	IIB	3	Moko	10
IIB4	IIB	4	Moko	4
IIB4NPB	IIB	4NPB	Epidemiological variant 4NPB	11
III	III	All Phylotype III sequevars: 19;20;21;22;23;29;42;43;49		14
IV	IV	All Phylotype IV sequevars: 8;9;10;11		15
RsIV	IV	8;9^a^;10^a^, 11		5
BDB	IV	10	*R. syzigii* subsp. *Celebensis*	5
SZY	IV	9^b^	*R. syzigii* subsp. *indoniensis*	5

a *Only R. solanacearum strains*.

b *Only SZY strains in seq 9*.

c *Number of hybridized strains used for validating each group of interest on the RsscAT*.

### Probe design and selection

The genomes used to design the probes are freely available on the MicroScope platform (available online at https://www.genoscope.cns.fr/agc/microscope/home/index.php; Vallenet et al., 2009; Genoscope, Evry, France) and the EMBL nucleotide sequence database (Ailloud et al., 2015). A first bioinformatics screening of candidate CDSs was performed using both the comparative genomics tool of the MicroScope platform and a BLAST search against NCBI databases to select CDSs that were conserved in the different phylogenetic subgroups and presented limited or absent identities with DNA sequences from non-target genomes present in the databases. A second bioinformatics selection eliminated the CDSs that were too close to mobile elements (e.g., transposases, integrases, etc.) using a parsing R (v3.0.0) script (R Development Core Team, 2011). The probes (1–9 per CDS) were designed using two software programs, the Array Oligo Selector (AOS; Bozdech et al., 2003) and OligoArray (OA; Rouillard et al., 2003), according to the following specifications: 50-mer length, 40–70% GC content, 2 probes per CDS, binding energy ranging between −20 and −35 kcal/mol (AOS), Tm 68–88°C (OA), exclusion of probes including 20 AT or more (AOS), and exclusion of probes including five consecutive repetitions of one of the four bases (OA). The probes were selected from a file that included all of the previously selected CDSs vs. a file that compiled all of the *R. solanacearum* genomes. A total of 256 candidate probes were generated, and their specificity was verified using a BLAST search against NCBI databases and a specific database that includes Rssc genomes and non-target genomes (strains genetically close to the Rssc or strains occurring in the same ecological niche (Supplementary Table 1). Primer pairs flanking the candidate probes were designed using Primer 3 (Untergasser et al., 2012), and PCR assays were performed with the selected strains representative of the Rssc diversity (Table 3) to validate the occurrence of the targeted DNA in the different subgroups. Following the PCR screening that removed the candidate DNA regions that were improperly amplified by the target strains, a batch of 100 probes was selected to be implemented and cribbed in the AT technology. This last selection removed probes that yielded unexpected results and generated the final set of 32 validated specific biological probes (Table 4), including a negative control probe that verifies the hybridization process. This set of 32 probes/primers was used to multiplex the 17 groups of interest (RsscAT).

**Table 4:** **Probes and associated primers of the RsscAT design**.

**Probe**	**Probe sequence (5′–3′)**	**Primer**	**Primer sequence**	**CDS**	**Nb hybridized strains**
GC_07	GGGTTATCACCTATGTAGAGTGCATAGATAAAAACAATCGAATTGGAAAG	759	GTCGCCGTCAACTCACTTTCC	*IpxC*	128
I_03	GGTATGCGAGGCGAATCCGGAGGCAGCGTTTCTGGTGAATGCCGAAGGCA	334	TCCTCCGCCGCCGACTTGCT	I_RSc3152 2R	7
I_05	GCGAGGCGAATCCGGAGGCAGCGTTTCTGGTGAATGCCGAAGGCACCTTG				
II_03	CGGGTCAAACCGGAAGATGAGAAGGACACGGCGCCGCCAAGTAGTGGCCG	459	GCCGATCGCCGGCCACTAC	II_RSK60v2_mp20181_3R	80
IIA_03	GAGTTGCAAAAGCTGCAGCAGAAGACGATACCGGTCGTGACACTCGACCC	352	CGAGGCGATCAACGGCGCGA	RSK60v2_mp30236	30
IIA24_04	ATTACGCACACGCGGCGGGCGTCAACCATACGACCCTGGTCAAGCACACA	463	TCAGCCGCCGTCGCTGTC	IIA24_RALB5v1_1210003_2R	9
IIA24_07	TGATGAACGGCGCGAGACAGACGCCTGGGAGCCGGTGATTGCTGAATGGT	465	ATCGTCGCGCACCCATCCTC	IIA24_RALB5v1_2390026_R	
IIA6_09	TACGATGACTTCAATCTGGCCGTGTTTATAGATGGCCCGCACCATGAGAG	475	TGCATTCGCGGCAAATACTGC	IIA6_RALUWv1_2330002_3R	7
IIA6_11	TGAGGACCTCGGCTACATCGTCGTGCGTTTTGCCAAAGAAATCACTAGCT				
IIA7_03	AAGAGAGTTGGATTCCAAACGATGCTGTACTACGCAAAGACCGTAGGCAG	363	TCGGAGCCTGTGTGCAGCGGA	IIA7_RSK60v2_130132 R	7
IIA7_11	CCAAGACCTGTTCCCGCAGGTTCTTTGTCCAAGTGCCACTTTTGAAGATG	371	GCCTGCCAAGGCGTCCAGCC	IIA7_RSK60v2_80203 2R	
IIB_5	GCAATGGACCGATTCCCGTCGGAACGTATTACATCCTGGATAGACCCTCC	ER	CCGTTGAACAGATCGCGGAA	IIB5_193R20	50
IIB_6	ATGTGGAGATGGCCTATGTTCTGTTCGTGGCATTTGCCAGCGTATCGATC	FR	CGTAAGATCGCCCGTCCC	IIB6_240R18	
IIB1_05	GCCAAATCGCCGTGCCGATGGTCAATGGTGACAACGGTTTCCACTTCGTA	631	ATTCACATGCAATTCGCCTAC	Genomic DNA fragment “prophage region”	24
IIB1_06	TGAAACTGCCCAGCAGGTCGCCATTCCCATACAGAATTCGACCGGCACGC				
IIB1_10	TACAACATCAGCACCAACGGCAATATCCTCTCGGGTGACATTTACCTGAA	AR	AGCGAAGTTGCCGGAGACGTAC	IIB8_436R18	
IIB1_12	AACGGCAATATCCTCTCGGGTGACATTTACCTGAACCCGGACCAGAACTT				
IIB3_08	GAATTTCGGAACGGTATCCTGAAGCAACCCTTTGGCGAAGCGTGCCTGCA	482	AATGGCTCGGCCCGCTCAAC	IIB3_RALMO_3796_R	10
IIB3_10	AAGAATTGACCGTTCCCTATGTCGTCTACTGGGAAGGTGAAGTACCAACG				
IIB4_04	TATACCGCAAGGGTATTCACCTGCGGGACAAAAGTCGCGATGCTGTTAGT	486	TGGGTGCAGAACAGGCGAACC	IIB4_RALW3v1_2510028_R	4
IIB4_08	GTCAAGGGGGCTGGCGATTTTGATATCTACGCTACCGCGCTCGCAGAGGC	488	CCTGGCCACGCGTCTTCGTT	IIB4_RALW3v1_2510028_2R	
IIB4NPB_16	CCGCTCCCATTCCTCCGCGTCTTTATTTCTGGGGGCACCTGCAGTTCCGC	494	TCACGGTTCCGGAAAATAGGACAG	IIB4NPB_RALBFv1_2920001_2R	11
IIB4NPB_35	CGGGGCGCAAGGATACCCAGCCGACCACGAAGTCCTCGAAAGGCCCGAGT	498	TCCGGTTGATCCGGCGGTAA	IIB4NPB_RALBFv1_840046_2R	
III_01	TCCGCGGACGTCCGGGTGACGCCTTGCTCGAGCCGGAATATGGCGACGGT	156	GCTCGTACGCCTGAATCTGT	IIICMR15v5_0623R	14
III_04	ATGGCGACGGTCACGCCTCACTCGAGGCGTTCCTCCGGGAGGAACAGATT				
IV_09	GTGCTCGACCAGGACAAGCTGAACACCGATCTGGATGCGGTCAAGGATTT	425	GCGACCGAATGCGGCGGTGT	IV_RPSI07_mp1774 R	15
IV_10	AGGACAAGCTGAACACCGATCTGGATGCGGTCAAGGATTTATCGCGCTTT				
RsIV_01	CGCGATCTACCTGTCCGCGTCCTACTACGAAAGATGGGCATTGGGAATCG	512	GGTAGGGCTCGAATGCCTCGAT	IV10_RPSI07_2728_2R	5
RsIV_16	GCTGCAGCACTGGTGGCGTAAGGATGAGTTGCCATTCCCAACCAAGGCGA	435	CGCACCTTGGCCGTGCGTGA	RsIV_RPSI07_p0011 R	
BDB_02	GTACATCGACCAGCAGTTCAGCAAGTCGGTACAGTTCCGCACCTCCTATC	449	GCGCGAATCTGGCGGAATGT	BDB_BDBv4_60028_2R	5
SZY_04	TGCATCCCGACACGCTCAGGGTTATCAAGCCGAACAAGGGGTTCGATAAG	521	GCGCGCATTTTGCTCACTCG	SZY_RALSYv4_11140_2R	5
NEG_Cupri07	CTGGCGCGCTTATCATCCGCGGCACCGTATTTCCGGGAACAGGAAAACGG	PR	CACGCATTCCCGACAAAT	na	128

The RsscAT were manufactured by Alere Technologies and consisted of oligonucleotide probes with a 3′ amino modification and C6 spacer. The probes were spotted in duplicate (first batch—AT1) along with other non-specific probes that were not analyzed; and the probes were also spotted in triplicate (second batch—AT2).

### Multiplex linear DNA amplification and labeling

Strains were grown overnight in Luria-Bertani broth (LB) at 28°C with agitation at 250 rpm, and then mild centrifugation was performed at 5,100 rpm for 10 min at 10°C. To prepare the cells for a DNA extraction, the pellets were washed twice with 500 μL of 1 M NaCl solution, centrifuged at 5,100 rpm for 10 min at 10°C, and then processed using the Wizard Genomic DNA Purification Kit (Promega, Madison WI, USA) following the manufacturer's instructions.

Following this DNA extraction, a linear PCR amplification was performed in which only one reverse primer was used to linearly amplify each targeted sequence (Table 4). The labeling of the genomic DNA was accomplished during the linear amplification step by using dUTP linked biotin as a marker, which allowed site-specific internal labeling of the corresponding target region, thus leading to the production of single-stranded biotin-labeled products. A total of 25 individual reverse primers were used in a multiplex linear PCR amplification for each Rssc strain. Using the HybridisationPlus Kit (Alere Technologies), 5 μL of DNA normalized with 200 ng/μL of genomic DNA were labeled according to the manufacturer's recommendations (3.9 μL Labeling Buffer B1, 0.1 μL Polymerase B2) and 1.35 μM of each of the 25 primers. The linear PCR amplification was performed using a Veriti Thermal Cycler (Applied Biosystems, Carlsbad, CA, USA) with the following parameters: 5 min at 96°C; followed by 45 cycles of 60°C for 20 s, 72°C for 30 s, and 96°C for 20 s; and a final hold at 4°C.

### Microarray hybridization and data acquisition

The reagents required for the hybridization steps were provided by the HybridisationPlus Kit (Alere Technologies), and the protocols were performed according to the manufacturer's recommendations except for certain conditions that were optimized according to the probe design requirements.

The prewashing steps of the RsscAT consisted of adding 500 μL of 60°C preheated ultrapure water for a 5 min incubation at 60°C and stirring at 550 rpm. The flow was discarded, and then 200 μL of hybridization buffer C1 preheated at 60°C was added and incubated for 2 min at 60°C with stirring at 550 rpm. The flow was discarded, and then 100 μL of hybridization mix consisting of 10 μL of labeled DNA and 90 μL of the hybridization buffer (C1) preheated at 60°C was transferred into a prewashed RsscAT and then incubated for 1 h incubation at 60°C with stirring at 550 rpm.

The washing steps consisted of heating the hybridized RsscAT at 42°C and discarding the hybridization mix, and then 500 μL of preheated washing buffer 1 (C2) at 42°C was added and incubated for 5 min at 42°C with stirring at 550 rpm. The flow was then discarded, and these steps were repeated two more times, with the last repetition performed at 30°C.

The conjugate mix, which consisted of 1 μL of preheated HRP conjugate 100x (C3) and 99 μL of preheated conjugate buffer (C4) at 30°C, was added to the washed RsscAT and then incubated for 10 min at 30°C with stirring at 550 rpm. Another washing step consisted of discarding the flow, adding 500 μL of washing buffer 2 (C5), and then incubating for 5 min at 30°C with stirring at 550 rpm. The final coloration step consisted of discarding the flow, adding 100 μL of HRP substrate buffer (D1) that was already acclimated to room temperature, and incubating for 5 min at 30°C without stirring.

Data acquisition was performed with the software provided with the ATR03 scanner (Alere Technologies) following the manufacturer's instructions.

### Data analysis

Hybridization signals were processed using the IconoClust software version 3.3, and all of the spots were automatically normalized by the software according to the equation *NI* = 1 − *(M/BG)*, where *NI* is the normalized intensity, *M* is the average intensity of the automatically recognized spot, and *BG* is the intensity of the local background. The output range of the signals was between 0 and 1, with 0 being negative and 1 being the maximal possible signal value. These raw data were processed using a home-made R script (provided upon request) that could set a threshold to discriminate positive probe signals from negative probe signals. The threshold was manually assigned to 0.6, which allowed for clear discrimination of positive and negative signals. The threshold for the control probe GC_07 (Rssc strains) was set to 0.4. Signals with intensities higher than the assigned threshold were considered positive and set as “1.” Signals lower than the assigned threshold were regarded as negative and set as “0.”

### Statistics

Different replicates were performed to assess the repeatability of the protocol. Each probe was spotted in duplicate (batch AT1) or triplicate (batch AT2) to evaluate the intra-assay repeatability, which was calculated based on the consistency rate within each batch or for both batches. Several strains (2 for batch AT1 and 6 for batch AT2) were tested twice or three times in the same batch to evaluate the inter-assay repeatability, which was calculated based on the proportion of consistent results among the different hybridization repeats.

Twenty-seven strains were tested in both the AT1 and AT2 batches. For these strains, the relative specificity, $SP=\frac{TN}{TN\;+\;FP}$; the relative sensitivity, $SE=\frac{TP}{TP\;+\;FN}$; and the Diagnostic Odds Ratio, $DOR=\frac{TP/FP}{FN/TN}$ and the corresponding 95% confidence interval (95% CI), were calculated for each batch, where TP, FP, FN, and TN refer to true positive, false positive, false negative and true negative, respectively. The DOR value ranged from 0 to infinity, with higher values indicating better discriminatory test performance. The DOR values were compared between the two batches using the Breslow–Day test of the Homogeneity of Odds Ratios (*p* = 0.05).

Finally, the SP and SE were calculated for each group of interest using the data acquired for the 75 target strains and the 12 outgroups in the two batches.

## Results and discussion

The RsscAT was developed to evaluate diverse bacterial strains and represents an affordable, accurate, fast, and user-friendly diagnostic tool with a high potential for standardization and routine diagnostic testing use. Microarrays developed using the conventional glass slide format with fluorescence detection (e.g., Cy3/Cy5) have a high operational burden because of the high laboratory skill required to handle the material and perform the bioinformatics computations, as well as the high cost of the equipment. Although high-density microarrays are more likely to meet research laboratory expectations, routine laboratory diagnostics require more user-friendly and efficient protocols. AT format microarrays provide a valuable option for diagnosticians who need to efficiently test a large number of markers at the same time. The ability of the AT microarray to identify and discriminate strains of the Rssc was evaluated using a set of 75 target strains representing the 17 main groups of the Rssc and 12 outgroup strains that are found in the same environment and show a high likelihood of interfering with the identification of Rssc strains (pathogenic or saprophytic strains).

### Probe design

The first step in developing the RsscAT was to design accurate probes specific to the 17 phylogenetic groups of the Rssc. This design step was performed in several stages during which our strong focus on achieving the specificity of the selected templates (CDS or probes) was maintained via *in silico* analyses and experimental data.

Probes were designed with a 50-mer length constraint, which provided sufficient length for a probe that balances specificity and flexibility and is capable of identifying the best sequences markers (Palmer et al., 2006).

The probability of secondary structures (e.g., hairpins) was checked during the probe design and used to select candidate probes and then enhance the probe signals. Additionally, the use of single-stranded DNA as a template prevented competition between the probe and antisense strand and increased the probability of single-stranded DNA hybridization to the probe.

To our knowledge, we have designed the first probe set that can characterize Rssc ecotypes and strains regardless of the array technology, including macroarrays, microarrays, glass slides, nylon, or AT. Although various strain typing designs used in the medical field provide detection at levels below the species level (Schmoock et al., 2011; Braun et al., 2012), most of the designs available for Rssc strains have focused on detection at the species level (Aittamaa et al., 2008; Pelludat et al., 2009; Dobnik et al., 2014); moreover, these designs are constrained by the sensitivity of the tests, which is a drawback of array technology. Here, RsccAT was presented as a characterization protocol, and it can be integrated at the second step of strain identification after the steps for isolating and detecting the target Rssc strain (by ELISA, PCR, etc.). Therefore, the sensitivity constraint is not applicable because a significant amount of DNA can be extracted from the pure culture strains.

### Control responses

The internal positive controls from Alere Technologies generated the expected positive results, and the negative control probe NEG_Cupri07 generated the expected negative results for each hybridization. The control probe GC_07 for the Rssc strains generated the expected results for most of the strains; however, the following 4 phylotype III strains showed a lower intensity signal: 447, 594, MG464 (Madagascar), and CFBP4964 (Reunion). This result suggested the presence of mismatches between the probe and the amplified targets. Because a signal was observed between the expected positive and expected negative strains, we lowered the threshold for this specific probe to 0.4. Using this threshold, all of the strains of the Rssc tested positive (100% inclusivity). This last result was expected because the probe GC_07 is included in the UDP-3-O-acyl-GlcNAc deacetylase (lpxC) gene, which has been successfully used in the design and validation of specific primers for the Rssc (Opina et al., 1997; Villa et al., 2003; Fegan and Prior, 2005). Additionally, the 12 outgroup strains did not hybridize on any of the 32 developed probes except the *R. pickettii* strain LMG5942, which was detected by the probes III_04 and BDB_02 (83.3% exclusivity). Nevertheless, this false positive data was not considered because the *R. solanacearum* positive control probe GC_07 did not test positive for this strain. The results obtained with the control probes confirmed the reliability of the hybridization step and supported the high specificity of the RsscAT.

No significant results were observed between the odds ratios for the batches (Breslow–Day test, *p* = 0.4375), which indicated that the different strains presented globally similar responses when the two batches were tested; however, the common probes of the two arrays were spotted under different conditions for these two batches (different locations and repetitions), which indicates the high reproducibility of this AT microarray protocol.

The ability of the AT microarray to identify and discriminate strains of the Rssc was evaluated using a set of 75 target strains representing the 17 main groups of the Rssc and 12 outgroup strains that are found in the same environment and show a high likelihood of interfering with the identification of Rssc strains (pathogenic or saprophytic strains).

### Phylotype identification

High relative specificity of 98.07% (95% CI 96.65–99) and sensitivity values of 100% (95% CI 90.26–100) were obtained for both probes that typed phylotype I. Indeed, the probes designed to detect phylotype I yielded the expected specific positive results for all of the tested strains of phylotype I, and they only yielded a false positive signal for the strains CFBP1418 (IIB-4NPB) and JT663 (SZY).

Moreover, a relative specificity of 100% (95% CI 97.20–100) was obtained for all of the probes typing phylotype II. However, the relative sensitivity value (67.61–95% CI 60.87–73.84) indicated that these probes presented issues with inclusivity. The phylotype II probes could not accurately detect the Moko IIB-3 strains, IIB-4 strains, IB-4NPB strains and certain IIA strains (CIP239 and JQ1143). One of the repetitions did yield a positive signal for IBSBF1503 (IIB-4NPB), LNPV24.25 (IIB-4NPB), and MolK2 (IIB-3); however, full specificity was not observed.

However, high relative specificity and sensitivity values were obtained for the probes that detected phylotypes IIA and IIB, with 99.24% (95% CI 97.27–99.91) and 100% (95% CI 99.12–100) relative specificity for phylotypes IIA and IIB, respectively, and 94.94% (95% CI 87.54–98.60) and 97.81% (95% CI 95.29–99.19) relative sensitivity for phylotypes IIA and IIB, respectively. Thus, the target strains for phylotypes IIA and IIB were successfully detected except for strain CIP239 belonging to IIA-40.

All the strains from phylotype III were successfully detected by the two dedicated probes, and a 100% relative sensitivity value (95% CI 95.49–100) was observed. A high relative specificity value of 99.49% (95% CI 98.51–99.89) was also obtained, and false positive signals were only registered for probe III_04 when testing LMG 5942 (see Section Controls).

All of the phylotype IV target strains except strain JT663 were detected, and false positive values were not registered, thereby resulting in a 100% relative specificity value (95% CI 99.38–100) and a high relative sensitivity value of 93.67% (95% CI 85.84–97.91).

To conclude, the specific probes yielded the expected results and showed good specificity. The only group that showed lower reliability was the phylotype II probes, which did not yield true positive signals for the Moko IIB strains and epidemiological IIB-4NPB strains. The phylogenetic and genomic characteristics of the Moko IIB-4 strains and IIB-4NPB strains are closely correlated (Ailloud et al., 2015), whereas these characteristics of the Moko IIB-3 strains are less correlated. The lack of sensitivity of the phylotype II probes for this particular group of strains was not predicted by the computer analysis or PCR amplification of the target. Nevertheless, the results obtained with the probes targeting the two subgroups IIA and IIB inside phylotype II largely overcame the inclusivity problems shown with the phylotype II probes.

### Ecotype and lineage identification

The ecotype of the Rssc strains (e.g., brown rot, Moko, NPB, BDB, and RSY) is the lowest level of genetic diversity that can be detected with the RsscAT, and an accurate ecotype analysis must be performed with additional groups of probes. Hence, a hierarchical analysis of the probe groups must be performed to develop an accurate characterization of the target strain. Once the status of the Rssc strain is validated, the phylotype can be determined and then, if possible, the ecotype is also determined accordingly to a particular ecotype. This process can circumvent the interpretation of false negative/positive signals.

The results (Table 5) showed that the Grandville wilt ecotype from phylotype IIA-7, the epidemiological variant IIB-4NPB, and the BDB ecotype were fully and specifically detected.

**Table 5:** **Ecotype characterization results**.

**Ecotypes and lineages**	**General remarks**	**False positive responses**	**False negative responses**	**Relative specificity (%) (95%CI)**	**Relative sensitivity (%) (95%CI)**
IIA6	All Moko IIA-6 strains were detected			100 (99.41–100)	100 (92.89–100)
IIA7	All IIA-7 strains were detected			100 (99.43–100)	100 (90.26–100)
IIA24	All Moko IIA-24 strains were detected, except B26	IIA24_04 for strain Molk2 (Moko IIB-3) on one repetition out of two	Strain B6 not detected by both IIA24_04 and IIA24_07	99.84 (99.10–100)	77.78 (60.85–99.88)
IIB1-2	All strains Brown rot IIB-1 IIB-2 strains were detected	IIB1_10 and IIB1_12 for strain CFBP6783 (IIB-4NPB); for strain CMR15 (III)	IIB1_05 and IIB1_06 for strain CFBP3858; IIB1_10 and IIB1_12 for strain JT511	96.10 (94.8–97.15)	95.61 (92.08–97.88)
IIB3	4 out of 6 strains were detected		IMI370184 and JT644 not detected by IIB3_10	100 (99.31–100)	80.43 (66.09–90.64)
IIB4	All Moko IIB-4 strains were detected	IIB4_04 and IIB4_08 for strain DGBBC1138 (III)	CFBP1418 not detected by IIB4_04	99.08 (98–99.66)	76.92 (56.35–91.03)
IIB4NPB	All IIB-4NPB strains were detected	IIB4NPB_35 for strain CFBP4963 (III); IIB1_10 and IIB1_12 for strain CFBP6783 (IIB-4NPB)		99.84 (99.11–100)	100 (94.04–100)
RsIV	All RsIV strains were detected, except MAFF301558		MAFF301558not detected by RsIV_01 et RsIV_16, CFBP6727 not detected by RsIV_16	100 (99.43–100)	73.08 (52.21–88.43)
BDB	All BDB strains were detected	BDB_02 for strain CFBP1416 (IIB-3)		94.87 (91.81–97.04)	100 (75.29–100)
SZY	All SZY strains were detected, except JT663	SZY_04 for strain CFBP6727 (RsIV)	JT663 not detected by BDB_02	99.07 (97.31–99.81)	76.92 (46.19–94.96)

Brown rot strains were fully characterized by the dedicated specific probes by considering the complementary responses displayed by the four probes. Indeed, false negative responses obtained for the probes IIB1_05 and IIB1_06 were overcome by the IIB1_10 and IIB1_12 responses and vice versa. This result highlighted the importance to have several probes for a given group of interest, located if possible on different CDS, which was the case for the Brown-rot strains-targeting probes. Interestingly, the IIB1_10 and IIB1_12 probes matched with a specific genome region selected with a different *in silico* approach and used to develop a Brown rot strains specific real-time quantitative PCR assay (Stulberg and Huang, 2015).

The paraphyletic Moko strains are sorted into different clusters within phylotype II (IA-6, IIA-24, IIB-3, and IIB-4), which highlights the genomic complexity and heterogeneity of this ecotype. Although these strains are epidemiologically adapted to wilt *Musaceae*, these strains also show pathogenicity to *Solanaceae* in controlled conditions (Cellier and Prior, 2010). Moko sequevars 4 and 6 were specifically detected, whereas sequevars 3 and 24 showed false negative signals despite the amplification of the respective target sequences by PCR. The hybridization process between the probe and the amplified DNA from these three strains might have been compromised by substitution of the DNA sequence, thereby leading to false negative signals. The hybridization parameters have been fully discussed in Tomlinson et al. (2014). Several site substitutions at specific regions of the amplified DNA can lead to a significant decrease in the positive signal, and the same false negative characterization at the ecotype level was observed for strain JT663 in the SZY ecotype and MAFF301558 in the RsIV ecotype. Complementary specific PCRs could be used when information at the ecotype level is lacking (Table 1).

Several false negative signals were obtained for some ecotypes; however, as previously noted, a hierarchical analysis of the probe groups could help to resolve such strain assignments. For example, strain CMR15 has been typed on AT as an IIB-1 strain by the IIB1_06 probe; however, this strain has been identified as a phylotype III strain and could not be an IIB-1 strain.

Additionally, strain CFBP6783 has been identified as a probable IIB-1 strain; however, it belongs to the IIB-4NPB group. Nevertheless, this result is not reliable (non-homogeneous repetitions of two IIB-1 probes and no identification by the two other IIB-1 probes) because all IIB-4NPB probes indicate that this strain is actually an IIB-4NPB strain.

### Intra-repeatability, inter-repeatability, and reproducibility

A consistency rate of 99.65% was obtained for the duplicate spotted probes for the AT1 batch and 99.97% was obtained for the triplicate spotted probes in the AT2 batch; thus, the RsscAT method showed a high degree of intra-array repeatability. In addition, high inter-array repeatability values were also observed, with consistency rates of 97.06% (batch AT1) and 99.51% (batch AT2). For the set of 27 strains analyzed from both batches (AT1 & AT2), the major indicators of diagnostic test performance presented high values. Thus, the relative specificity, relative sensitivity, odds ratios and the corresponding 95% confidence intervals were 98.81% (95% CI 98.16–99.27), 96.07% (95% CI 93.08–98.02), and 1976.8 (95% CI 914.8 4300.3), respectively, when the strains were hybridized on batch AT1; and 98.81% (95% CI 98.36–99.17), 94.47% (95% CI 92.25–96.21), and 1371.37 (95% CI 869.92–2474.9), respectively, when the strains were hybridized on batch AT2.

No significant results were observed between the odds ratios for the batches (Breslow–Day test, *p* = 0.4375), which indicated that the different strains presented globally similar responses when the two batches were tested; however, the common probes of the two arrays were spotted under different conditions for these two batches (different locations and repetitions), which indicates the high reproducibility of this AT microarray protocol.

## Conclusion

The design presented here is highly reliable, and the repetitions of probes as well as the different levels of characterization were able to differentiate among the target strains within the 17 different groups of high importance within the Rssc. This design is also able to detect phylogenetic incongruence among strains according to the host isolation, and then can also detect emergent strains that will require further confirmation tests. Different outcomes and scenarios could emerge from this RsccAT analysis: for example, strains characterized as Moko on tomato (*Solanum lycopersicum*) represent serious cases and require immediate action; however, tests that rely on species-level analyses would give the positive result for the detection of “*R. solanacearum*” strains and a negative result for the specific detection of brown rot strains. Additionally, the ecotype level represents strain groups that are epidemiologically active worldwide; hence, the strains identified at the phylotype level but not at the ecotype level represent anomalies that require further investigation.

Currently, only limited eradication and containment strategies are available to control Rssc strains once they have been introduced into a territory. Hence, preventing such introductions and developing tools to identify and characterize the quarantined organisms remain priorities for the agricultural industry. The developed RsscAT dedicated to identifying Rssc strains was designed to minimize time and costs and maximize marker multiplexing and reliability, and it is only applied on pure culture strains. Multiplexing the markers into a compact, affordable, and user-friendly technology for infra-species-level characterizations of Rssc strains is the goal of the RsccAT design. A total of 17 groups can be interpreted in a phylogenetic hierarchical fashion that covers the main phylogenetic groups and ecotypes of the Rssc. Although ELISA, conventional or real time PCR, LAMP-PCR, and other molecular tools are designed to test for a multitude of strains in one reaction based on one or very few markers, the RsscAT is design to test for a multitude of markers in one reaction for one strain. This profile makes the RsscAT as a strong complementary diagnostic protocol within a diagnostic scheme. The RsscAT diagnostic array presents a number of benefits, such as an industry-standard format and a high level of reliability, and it also provides diagnostic laboratories with a user-friendly, cost-efficient and time-efficient option that can be used for epidemiological monitoring of Rssc strains.

## Author contributions

Conceived and designed the experiments: GC, IR, PP. Performed the experiments: GC, SA. Analyzed the data: GC, IR, SA, FC. Contributed reagents/materials/analysis tools: GC, IR, PP. Wrote the paper: GC, IR, PP.

### Conflict of interest statement

The authors declare that the research was conducted in the absence of any commercial or financial relationships that could be construed as a potential conflict of interest.
