# Supplementary material for: Tube-Wise Diagnostic Microarray for the Multiplex Characterization of the Complex Plant Pathogen Ralstonia solanacearum
Source: Front Plant Sci. 2017 May 24;8:821. doi: 10.3389/fpls.2017.00821 (PMC5442206; doi:10.3389/fpls.2017.00821)
Supplement: Supplementary file 1 [file DataSheet1.DOCX]

Supplementary Material

**Tube-wise diagnostic microarray for the multiplex characterization of the complex plant pathogen *Ralstonia solanacearum***

**Gilles Cellier^*^, Sandrine Arribat, Frédéric Chiroleu, Philippe Prior, Isabelle Robène**

*** Correspondence:** Gilles Cellier: [gilles.cellier@anses.fr](mailto:gilles.cellier@anses.fr)

| **Strain Name** | **Replicon** | **Reference** |
| --- | --- | --- |
| *Burkholderia* sp. 383 | Chromosome | Bcep18194_A NC_007510 |
| *Burkholderia* sp. 383 | Chromosome | Bcep18194_B NC_007511 |
| *Burkholderia* sp. 383 | Chromosome | Bcep18194_C NC_007509 |
| *Pseudomonas syringae* pv. *tomato* DC3000 | Chromosome | PSPTO NC_004578 |
| *Burkholderia cenocepacia* AU 1054 | Chromosome | Bcen_ NC_008060 |
| *Burkholderia cenocepacia* AU 1054 | Chromosome | Bcen_ NC_008061 |
| *Burkholderia cenocepacia* AU 1054 | Chromosome | Bcen_ NC_008062 |
| *Burkholderia mallei* ATCC 23344 | Chromosome | BMA NC_006348 |
| Burkholderia mallei ATCC 23344 | Chromosome | BMAA NC_006349 |
| *Burkholderia pseudomallei* K96243 | Chromosome | BPSL NC_006350 |
| *Burkholderia pseudomallei* K96243 | Chromosome | BPSS NC_006351 |
| *Burkholderia rhizoxinica* HKI 454 | Chromosome | RBRH_ NC_014718 |
| *Burkholderia rhizoxinica* HKI 454 | Chromosome | RBRH_ NC_014722 |
| *Burkholderia* sp CCGE1002 | Chromosome | BC1002_ NC_014117 |
| *Burkholderia* sp CCGE1002 | Chromosome | BC1002_ NC_014118 |
| *Burkholderia* sp CCGE1002 | Chromosome | BC1002_ NC_014119 |
| *Burkholderia* sp CCGE1002 | Plasmid | BC1002_ NC_014120 |
| *Burkholderia thailandensis* E264 | Chromosome | BTH_I NC_007651 |
| *Burkholderia thailandensis* E264 | Chromosome | BTH_II NC_007650 |
| *Burkholderia vietnamiensis* G4 | Chromosome | Bcep1808_ NC_009254 |
| *Burkholderia vietnamiensis* G4 | Chromosome | Bcep1808_ NC_009255 |
| *Burkholderia vietnamiensis* G4 | Chromosome | Bcep1808_ NC_009256 |
| *Burkholderia vietnamiensis* G4 | Plasmid | Bcep1808_ NC_009226 |
| *Burkholderia vietnamiensis* G4 | Plasmid | Bcep1808_ NC_009227 |
| *Burkholderia vietnamiensis* G4 | Plasmid | Bcep1808_ NC_009228 |
| *Burkholderia vietnamiensis* G4 | Plasmid | Bcep1808_ NC_009229 |
| *Burkholderia vietnamiensis* G4 | Plasmid | Bcep1808_ NC_009230 |
| *Burkholderia xenovorans* LB400 | Chromosome | Bxe_A NC_007951 |
| *Burkholderia xenovorans* LB400 | Chromosome | Bxe_B NC_007952 |
| *Burkholderia xenovorans* LB400 | Chromosome | Bxe_C NC_007953 |
| *Pseudomonas fluorescens* Pf5 | Chromosome | PFL NC_004129 |
| *Ralstonia eutropha* H16 | Chromosome | H16_A NC_008313 |
| *Ralstonia eutropha* H16 | Chromosome | H16_B NC_008314 |
| *Ralstonia eutropha* H16 | Plasmid | PHG NC_005241 |
| *Ralstonia eutropha* JMP134 | Chromosome | Reut_A NC_007347 |
| *Ralstonia eutropha* JMP134 | Chromosome | Reut_B NC_007348 |
| *Ralstonia eutropha* JMP134 | Plasmid | Reut_C NC_007336 |
| *Ralstonia eutropha* JMP134 | Plasmid | Reut_D NC_007337 |
| *Ralstonia pickettii* 12J | Chromosome | Rpic_ NC_010678 |
| *Ralstonia pickettii* 12J | Chromosome | Rpic_ NC_010682 |
| *Ralstonia pickettii* 12J | Plasmid | Rpic_ NC_010683 |

**Supplementary Table 1.** List of non-target genomes used for the specificity check by BLAST for the 256 candidate probes.
